# A Comparison of the Cheater Detection and the Unrelated Question Models: A Randomized Response Survey on Physical and Cognitive Doping in Recreational Triathletes

**DOI:** 10.1371/journal.pone.0155765

**Published:** 2016-05-24

**Authors:** Hannes Schröter, Beatrix Studzinski, Pavel Dietz, Rolf Ulrich, Heiko Striegel, Perikles Simon

**Affiliations:** 1 Department of Psychology, Eberhard Karls University Tübingen, Tübingen, Germany; 2 German Institute for Adult Education, Bonn, Germany; 3 Department of Sports Medicine, Johannes Gutenberg University Mainz, Mainz, Germany; 4 Department of Physical Activity and Public Health, Karl Franzens University Graz, Graz, Austria; 5 Department of Sports Medicine, Eberhard Karls University Tübingen, Tübingen, Germany; Kent State University, UNITED STATES

## Abstract

**Purpose:**

This study assessed the prevalence of physical and cognitive doping in recreational triathletes with two different randomized response models, that is, the Cheater Detection Model (CDM) and the Unrelated Question Model (UQM). Since both models have been employed in assessing doping, the major objective of this study was to investigate whether the estimates of these two models converge.

**Material and Methods:**

An anonymous questionnaire was distributed to 2,967 athletes at two triathlon events (Frankfurt and Wiesbaden, Germany). Doping behavior was assessed either with the CDM (Frankfurt sample, one Wiesbaden subsample) or the UQM (one Wiesbaden subsample). A generalized likelihood-ratio test was employed to check whether the prevalence estimates differed significantly between models. In addition, we compared the prevalence rates of the present survey with those of a previous study on a comparable sample.

**Results:**

After exclusion of incomplete questionnaires and outliers, the data of 2,017 athletes entered the final data analysis. Twelve-month prevalence for physical doping ranged from 4% (Wiesbaden, CDM and UQM) to 12% (Frankfurt CDM), and for cognitive doping from 1% (Wiesbaden, CDM) to 9% (Frankfurt CDM). The generalized likelihood-ratio test indicated no differences in prevalence rates between the two methods. Furthermore, there were no significant differences in prevalences between the present (undertaken in 2014) and the previous survey (undertaken in 2011), although the estimates tended to be smaller in the present survey.

**Discussion:**

The results suggest that the two models can provide converging prevalence estimates. The high rate of cheaters estimated by the CDM, however, suggests that the present results must be seen as a lower bound and that the true prevalence of doping might be considerably higher.

## Introduction

Recent surveys concerning substance use in sports indicate an alarming prevalence of the use of drugs enhancing physical and cognitive performance in both elite and recreational athletes [1–5]. For example, Dietz et al. [6] surveyed approximately 3,000 triathletes with an anonymous questionnaire and reported 12-month prevalences of 13.0% for physical doping (the use of illicit or banned substances to enhance physical performance), and 15.1% for cognitive doping (the use of illicit or prescription substances to enhance cognitive performance, e.g. vigilance, attention, concentration or memory) [7], respectively. These prevalences were estimated by indirect questioning in order to protect privacy, thereby encouraging honest responses [8]. Similar alarming results for physical doping were reported by Pitsch et al. [9] when surveying athletes of different disciplines with indirect questioning.

Compared to direct questioning, these indirect methods typically yield higher prevalence rates for sensitive issues and thus provide a more valid picture of behavior [10]. In a seminal paper, Warner [11] proposed the randomized-response technique to eliminate evasive answer biases. Participants are asked to answer one of two statements with “true” or “not true” (e.g., Statement A: I have used doping substances; Statement B: I never have used doping substances) and a randomization device (e.g., the hidden roll of a die) determines which statement is selected (e.g. A: numbers 1–4, B: numbers 5–6). Crucially, only the respondent knows the outcome of the randomization process. Thus, the specific statement she or he answers is hidden from the experimenter. Although it is impossible to reveal the sensitive attribute of a specific respondent, the experimenter can nevertheless estimate the prevalence of the attribute by aggregating the individual results across the entire sample. Since this original paper, many alternative randomized-response techniques have been suggested which try to optimize the statistical and psychological properties of this method [8].

The present study is a follow-up three years after the survey conducted by Dietz et al. [6] conducted on triathletes. Specifically, it aimed to address three objectives. First, we included a different randomized-response model to assess the reliability of this technique. The original study employed the Unrelated Question Model (UQM) [12]. In the present study, we mainly used the Cheater Detection Model (CDM) [13]. This model has been used by Pitsch et al. [9] to estimate the prevalence of physical doping. The UQM and the CDM have distinct advantages [14]. The UQM requires a smaller sample size and seems psychologically more acceptable than CDM [15,16]. By contrast, the CDM allows one to estimate not only a lower bound but also an upper bound of prevalences [13]. Despite these differences between the two models, it is important to know whether their estimates converge. Therefore, we also used the UQM for a subsample of the present study. If the estimates of these two models converge, this would indicate that each of the two models is capable to provide a realistic estimate of the prevalence rate. By contrast, if the estimates diverge, then at least one of the two models would fail to reflect the real frequency of doping.

The second objective was whether the prevalence rates are modulated by competition motivation. On a first glance, it seems reasonable that highly competitive athletes are especially prone to use doping substances[17,18]. Surprisingly, however, a previous study has shown that competitiveness does not predict doping behavior [19]. Thus, it seems important to reveal the generality of this finding. We therefore assessed the level of competition motivation by the corresponding scale of the Achievement Motivation Inventory (AMI) [20].

Finally, we were interested whether the prevalence rates for physical and cognitive doping changed since the previous survey of Dietz et al. [6]. We surveyed triathletes at the Ironman European Championship in Frankfurt and the Ironman European Championship 70.3 in Wiesbaden. Both Championships were also surveyed in the original study, which will allow a direct comparison of this follow-up study and the original study. The rationale for investigating physical and cognitive doping in a collective of triathletes is based on the fact that triathlon and especially full-and half-distance triathlon is an ultra-endurance sport. Therefore, persons that use substances to enhance physical performance in completion and training may also use cognitive-enhancing substances to enhance their workplace performance [6].

## Material and Methods

### Samples, Survey Procedure, and Ethics

The Ethics Committee of the Medical faculty, University of Tübingen, gave its approval for conducting this study. Participants were informed about the aim of the study and that their participation was anonymous and voluntary. The study surveyed non-professional, recreational athletes who participated in the long distance triathlon in Frankfurt (6^th^ of July 2014, European Championship, 3.8–180–42.2 km) and the half-distance triathlon in Wiesbaden (10^th^ of August 2014, European Championship 70.3, 1.9–90–21.1 km).

A single double-paged self-report paper-and-pencil-questionnaire was distributed to the participants. This distribution took place during the registration procedure in the race office on Thursday, Friday, and Saturday and thus prior to the competition on Sunday. Following the completion of the questionnaire, participants were asked to drop the questionnaire into a black box. The questionnaire did not ask for any detailed personal information (e.g., name, address, exact date of birth) to further enhance objective and subjective anonymity. Participants were asked whether they preferred to receive the questionnaire in German or in English language.

### Questionnaire

The beginning of the questionnaire’s front side provided information about the aim of the study and the responsible institution (Department of Sports Medicine, Prevention and Rehabilitation Division, Johannes Gutenberg University Mainz). Furthermore, athletes were informed that their participation was anonymous and voluntary. Next, participants were asked to answer 10 items assigned to the competition dimension of the Achievement Motivation Inventory (AMI) by [20] (original German version: Leistungsmotivationsinventar) [21]. Each item had to be answered according to a 7-level Likert-scale (ranging from “1: strongly disagree” to “7: strongly agree”) by marking one of the seven corresponding squares.

On the beginning of the questionnaire’s back page, participants were asked whether they had consumed physically or cognitively enhancing drugs, respectively, within the last 12 months. The sensitive questions as well as the descriptions for physical and cognitive doping were identical to those in Dietz et al. [12]. Specifically, the sensitive question about physical doping was: “Have you taken substances to increase your physical performance in the past 12 months that are only available at a pharmacy, at the doctor’s office or on the black market (e.g. anabolic steroids, EPO, growth hormones, stimulants)?” and the sensitive question about cognitive doping was: “Have you take substances to increase your mental performance in the past 12 months that are only available at a pharmacy, at the doctor’s office or on the black market (e.g. caffeine pills, stimulants, Cocaine, Methylphenidate / Ritalin®, beta blocker, Modafinil)?”

Anonymity was ensured by means of randomized response techniques, the details will be outlined in the next section. The order of the sensitive questions about physical doping and cognitive doping, respectively, was counterbalanced across participants. At the end of the questionnaire participants were asked to provide information about their gender, age, body height, body weight, years of training, hours of training per week, kilometers of training per week (separately for swimming, cycling, and running), whether or not they did A-levels (i.e., whether they had university entrance qualification), whether or not they mainly train within a training group, and whether they participate in the triathlon as a solo athlete or as a member of a relay team.

### Randomized Response Techniques

#### Cheater Detection Model (CDM)

Prevalence rates for physical and cognitive doping for the sample surveyed in Frankfurt and for one subsample surveyed in Wiesbaden were assessed by the CDM. For details of this method see [13,16]. In general, a neutral question determined whether participants were directed to the sensitive question or simply should respond with “yes”. Depending on the neutral question, participants were directed to the sensitive question either with probability *p*_1_ = 0.25 (50% of all participants) or with the probability *p*_2_ = 0.75 (remaining 50% of participants). Participants, who were directed to the sensitive question about physical doping with *p*_1_, were directed to the sensitive question about cognitive doping with *p*_2_ and vice versa.

The neutral question asked about the birthday of the athlete’s mother or the birthday of the athlete’s best friend (*p* = .25: “Is it in the first three months of a year (January–March)?”; *p* = .75: “Is it in the last nine months of a year (April–December)?”). If the answer to the neutral question—that was only known to the athlete—was affirmative, participants were instructed to answer the sensitive question truthfully, whereas they were instructed to simply respond “yes” otherwise. The assignment of the two neutral questions (i.e., about the birthday of the mother or the birthday of the best friend) to the two sensitive questions was counterbalanced across participants.

According to CDM, for the critical question the proportions of honest-yes respondents (honest dopers), honest-no respondents (honest non-dopers), and cheaters (athletes who ignore the procedural instructions and always respond with “no” irrespectively of whether they are dopers or non-dopers) are *π*_*S*_, *β*, and *γ*, respectively, with *π*_*S*_ + *β* + *γ* = 1. Note, that *π*_*S*_ represents a lower bound for the proportion of dopers, and that the effective proportion may be as high as *π*_*S*_ + *γ* in case that all of the cheaters would be dopers. Formula for estimating these parameters and the corresponding standard errors are given by [13].

#### Unrelated Question Model (UQM)

To allow for a comparison with the survey of Dietz et al. [12], prevalence rates for physical and cognitive doping for the one subsample surveyed in Wiesbaden was assessed by the UQM. In general, a neutral question determined whether participants were directed to the sensitive question or to a non-sensitive question. The neutral question asked whether the birthday of the athlete’s mother (50% of all participants) or the birthday of the athlete’s best friend (remaining 50% participants) is within the first ten days of a month. In case of an affirmative answer—that was only known to the athlete—, he/she was instructed to answer the non-sensitive question. Otherwise, the athlete was instructed to answer the sensitive question.

Therefore, the probability was *p* = 0.67 (1—*p* = 0.33) that a participant was directed to the sensitive (non-sensitive question). The non-sensitive question asked whether the birthday of the athlete’s mother (50% of all participants) or the birthday of the athlete’s best friend (remaining 50% of all participants) was in the first half of the year. This principal UQM procedure was used separately for assessing physical and cognitive doping.

Formula for estimating UQM’s parameter *π*_*S*_ (prevalence rate) and its associated standard error are given by [12]. In order to compare the estimates from CDM and UQM in the Wiesbaden sample, we computed a likelihood-ratio test by using a numerical search algorithm for optimizing the compound likelihood function of CDM and UQM. Under the null hypothesis H_0_, the prevalence estimates of the two procedures were constrained to be equal, whereas separate estimates were computed under the alternative hypothesis H_1_. Under H_0_, the likelihood-ratio is approximately χ^2^- distributed with *df* = 1 ([22], p. 441).

## Results

### Response Rate and Outlier Rejection

In total, 2,967 athletes participated in the survey and 2,209 athletes (74.45% of total sample) fully completed the questionnaire. The present total sample size was almost identical to the one in the study of Dietz et al. [6] and considerably larger than the sample sizes in other RRT surveys on doping [3,9]. The completed questionnaires were controlled for outliers separately for the three subsamples (Frankfurt, CDM; Wiesbaden, CDM; Wiesbaden, UQM). Questionnaires were excluded from data analysis if one or more values given for age, body height, body weight, years of training, hours of training per week, and kilometers of training per week (separately for swimming, biking, and running), was within the 0.5 or the 99.5 percentile of the sample, respectively. An additional visual inspection which followed the outlier rejection based on the percentile criterions, resulted in the exclusion of four questionnaires of the Frankfurt sample, because at least one of the filled in values for kilometers of training per week was exceptionally large (running > 129 km, biking > 550 km, swimming > 25 km). In total, 2,017 questionnaires (67.98% of total sample; 91.31% of fully completed questionnaires) were included in data analysis. Table 1 shows the number of participants in dependency of location and RRT method, the number of incompletely answered questionnaires, the number of fully answered questionnaires, and the number of questionnaires included in data analysis after outlier rejection.

**Table 1:** Number of participants, missing data, fully completed questionnaires, and included questionnaires.

	Location and RRT Method			
	Frankfurt (CDM)	Wiesbaden (CDM)	Wiesbaden (UQM)	Total
**Participants,** N (% of sample)	1,573 (100%)	699 (100%)	695 (100%)	2,967 (100%)
**Missing Data,** N (% of sample)	464 (29.50%)	170 (24.32%)	124 (17.84%)	758 (25.55%)
**Missing Bibliographical Data,** N (% of sample)	190 (12.09%)	46 (6.58%)	42 (6.04%)	278 (9.37%)
**Missing Training Data,** N (% of sample)	237 (15.07%)	119 (17.02%)	80 (11.51%)	436 (14.69%)
**Missing Solo Athlete / Relay Team Information,** N (% of sample)	106 (6.74%)	27 (3.86%)	26 (3.74%)	159 (5.36%)
**Missing Competition Motivation Data,** N (% of sample)	48 (3.05%)	20 (2.86%)	14 (2.01%)	82 (2.76%)
**Missing Answer to Sensitive Question about Physical Doping,** N (% of sample)	193 (12.27%)	50 (7.15%)	42 (6.04%)	285 (9.61%)
**Missing Answer to Sensitive Question about Cognitive Doping,** N (% of sample)	193 (12.27%)	54 (7.73%)	42 (6.04%)	289 (9.74%)
**Fully Completed Questionnaires,** N (% of sample)	1,109 (70.50%)	529 (75.68%)	571 (82.16%)	2,209 (74.45%)
**Included Questionnaires After Outlier Rejection,** N (% of sample)	1,001 (63.64%)	482 (68.96%)	534 (76.83%)	2,017 (67.98%)

### Bibliographical Data, Training Characteristics, and Competition Motivation of the Participants

Table 2 summarizes the bibliographical and training data for the three samples surveyed at Frankfurt (CDM) and Wiesbaden (CDM, UQM). In general, these data were virtually identically to the data of the samples surveyed by Dietz et al. in 2011 [6]. As in the previous study, most of the athletes were male (N = 1,781; 88.30% of included cases), and the mean age was 39.81 years (SD: ± 8.79; range 19–69). On average, athletes had a training experience of 9.97 years (SD: ± 8.27, range: 1–48) and trained 13.01 hours per week (SD: ± 4.59, range: 2.5–35).

**Table 2:** Bibliographical data, training data, and competition motivation.

	Location and RRT Method			
	Frankfurt (CDM)	Wiesbaden (CDM)	Wiesbaden (UQM)	Total
**Included Cases**	1,001	482	534	2,017
**Gender** (N)	88.31% male (884)	86.31% male (416)	90.07% male (481)	88.30% male (1,781)
	11.69% female (117)	13.69% female (66)	9.93% female (53)	11.70% female (236)
**Age,** years (mean; SD)	23–63 (40.88 years ± 8.15)	19–69 (38.37 years ± 9.31)	19–68 (39.11 years ± 9.22)	19–69 (39.81 years ± 8.79)
**Height,** cm (mean; SD)	152–198 (178.94 cm ± 7.91)	159–201 (179.43 cm ± 7.68)	161–198 (179.90 cm ± 7.38)	152–201 (179.31 cm ± 7.73)
**Weight,** kg (mean; SD)	49–100 (73.82 kg ± 8.94)	51–103 (73.60 kg ± 9.54)	50–100 (74.53 kg ± 9.45)	49–103 (73.95 kg ± 9.22
**BMI,** kg/m^2^ (mean; SD)	16–32 (22.99 kg/m^2^ ± 1,98)	18–30 (22.80 kg/m^2^ ± 2.04)	18–33 (22.98 kg/m^2^ ±2.14)	16–33 (22.94 kg/m^2^ ±2.04)
**A-Level** (N)	78.22% yes (783)	75.10% yes (362)	75.66% yes (404)	76.80% yes (1,549)
	21.78% no (218)	24.90% no (120)	24.34% no (130)	23.20% no (468)
**Questionnaire version** (N)	60.24% German (603)	75.10% German (362)	75.47% German (403)	67.82% German (1,368)
	39.76% English (398)	24.90% English (120)	24.53% English (131)	32.18% English (649)
**Years of training, years** (mean; SD)	1.5–40 (10.06 years ± 7.98)	1–35 (9.01 years ± 7.62)	1–48 (10.65 years ± 9.23	1–48 (9.97 years ± 8.27)
**Hours of training/week,** hours (mean; SD)	3–35 (13.62 hours ± 4.36)	3–27 (12.19 hours ± 4.53)	2.5–30 (12.61 hours ± 4.88)	2.5–35 (13.01 hours ± 4.59)
**Training kilometers/week,** km (mean; SD)—**Swimming**	0.20–25 (6.30 km ± 3.10)	0.50–20 (5.80 km ± 3.61)	0.10–20 (5.94 km ± 3.42)	0.10–25 (6.09 km ± 3.32)
**Training kilometers/week,** km (mean; SD)—**Biking**	15–550 (191.69 km ± 8.18)	20–450 (164.61 km ± 81.94)	7–500 (173.55 km ± 87.59)	7–550 (180.42 km ± 87.31)
**Training kilometers/week,** km (mean; SD)—**Running**	5.50–129 (43.08 km ± 18.10)	3–100 (37.96 km ± 18.24)	3–100 (39.35 km ±17.56)	3–129 (40.87 km ± 18.12)
**Mainly training in a training group** (N)	23.38% yes (234)	20.75% yes (100)	17.60% yes (94)	21.22% yes (428)
	76.62% no (767)	79.25% no (382)	82.40% no (440)	78.78% no (1,589)
**Participating as a solo athlete** (N)	98.30% (984)	99.59% (480)	99.25% (530)	98.86% (1,994)
**Participating as member of a relay team** (N)	1.70% (17)	0.41% (2)	0.75% (4)	1.14% (23)
**Competition motivation** Likert-sum (mean; SD; median)	10–70 (39.73; 10.01; 40)	10–70 (42.15; 10.30; 42)	11–70 (41.42; 10.93; 41)	10–70 (40.76; 10.38; 41)
**lowly competitive [≤ median]** (N) Likert-sum (mean; SD)	52.75% (528) 10–40 (32.14 ± 6.11)	50.62% (244) 10–42 (34.00 ± 6.50)	50.19% (268) 11–41 (32.64 ± 6.60)	51.56% (1,040) 10–42 (32.71 ± 6.37)
**highly competitive [> median]** (N) Likert sum (mean; SD)	47.25% (473) 41–70 (48.20 ± 5.85)	49.38% (238) 43–70 (50.51 ± 5.78)	49.81% (266) 42–70 (50.26 ± 6.33)	48.44% (977) 41–70 (49.32 ± 6.06)

Table 2 also contains the competition motivation data. The mean score for competition motivation was 40.76 (SD: ± 10.38, range: 10–70; a higher score indexes a higher competition motivation), which closely resembles the corresponding mean score of the AMI’s total norm sample (mean: 41.70 points, SD: ± 11.22, range: 10–70, [21]). We conducted a median-split analysis to investigate whether athletes with a higher competition motivation (i.e., with a score above the median) had different prevalence rates as compared to those with lower competition motivation (i.e., with a score on or below the median). Athletes in the former group had a mean score of 49.32 points and athletes in the latter group had a mean score of 32.71 points.

### Prevalences

Table 3 contains the estimated 12-month prevalences as a function of location, RRT method, and level of competition motivation. For the Frankfurt sample (CDM), the estimate of honest-yes respondents *π*_*S*_ (honest dopers) to the critical question concerning physical doping was about 12% but did not differ for lowly and highly competitive participants. The estimate of the cheater rate *γ* was larger than 60% indicating that many participants did hesitate to answer truthfully (saying “yes”). A similar pattern of results was obtained for cognitive doping with a prevalence rate of about 9% and a cheater rate of about 60%. Because of the high cheater rate, the true rate of dopers might be much larger than reflected by the rate of honest-yes respondents.

**Table 3:** Estimated 12-month prevalences as a function of location, method, and level of competition motivation.

	‘Yes’		‘No’		*a*		${\hat{\pi }}_{S}$	$Var({\hat{\pi }}_{S})$	95% CI (${\hat{\pi }}_{S}$)	$\hat{\beta }$	$Var(\hat{\beta })$	95% CI ($\hat{\beta }$)	$\hat{\gamma }$	$Var(\hat{\gamma })$	95% CI ($\hat{\gamma }$)
Location and Method	*p*_*1*_	*p*_*2*_	*p*_*1*_	*p*_*2*_	*p*_*1*_	*p*_*2*_									
**Frankfurt, CDM** (p_1_: 0.25; p_2_: 0.75; N_included_: 1001)															
Physical Doping															
all	144	90	348	419	0.293	0.177	11.9%	0.00075	6.5–17.3	23.2%	0.00283	12.8–33.6	64.9%	0.00102	59.6–70.3
lowly competitive	79	50	175	224	0.311	0.182	11.8%	0.00144	4.4–19.2	25.7%	0.00555	11.1–40.3	62.5%	0.00203	55.0–69.9
highly competitive	65	40	173	195	0.273	0.170	11.9%	0.00156	4.1–19.6	20.6%	0.00574	5.7–35.4	67.5%	0.00203	59.8–75.3
Cognitive Doping															
All	168	85	341	407	0.330	0.173	9.4%	0.00076	4.0–14.8	31.5%	0.00290	20.9–42.0	59.1%	0.00105	53.7–64.5
low competitive	88	45	186	209	0.321	0.177	10.5%	0.00149	3.0–18.1	28.8%	0.00548	14.3–43.3	60.7%	0.00193	53.1–68.2
highly competitive	80	40	155	198	0.340	0.168	8.2%	0.00156	0.4–15.9	34.5%	0.00617	19.1–49.9	57.3%	0.00230	49.6–65.1
**Wiesbaden,** CDM (p_1_: 0.25; p_2_: 0.75; N_included_: 482)															
Physical Doping															
all	89	34	161	198	0.356	0.147	4.2%	0.00144	-3.3–11.6	41.9%	0.00582	26.9–56.8	53.9%	0.00220	46.5–61.4
lowly competitive	41	19	89	95	0.315	0.167	9.2%	0.00316	-1.8–20.2	29.7%	0.01152	8.7–50.8	61.0%	0.00404	50.0–72.0
highly competitive	48	15	72	103	0.400	0.127	-0.9%	0.00262	-11.0–9.1	54.6%	0.01176	33.3–75.8	46.4%	0.00474	36.3–56.4
Cognitive Doping															
all	97	36	135	214	0.418	0.144	0.7%	0.00137	-6.6–8.0	54.8%	0.00617	39.4–70.2	44.5%	0.00248	37.2–51.7
lowly competitive	47	18	67	112	0.412	0.138	0.2%	0.00260	-9.8–10.1	54.8%	0.01217	33.1–76.4	45.1%	0.00501	35.1–55.1
highly competitive	50	18	68	102	0.424	0.150	1.3%	0.00291	-9.3–11.9	54.7%	0.01253	32.8–76.7	43.9%	0.00492	33.4–54.5
**Wiesbaden,** UQM (p_1_: 0.67; N_included_: 534)															
Physical Doping															
all	102	-	432	-	0.191	-	3.9%	0.00064	-1.1–8.9	-	-	-	-	-	-
lowly competitive	50	-	218	-	0.187	-	3.2%	0.00126	-3.7–10.2	-	-	-	-	-	-
highly competitive	52	-	214	-	0.195	-	4.5%	0.00132	-2.6–11.7	-	-	-	-	-	-
Cognitive Doping															
all	118	-	416	-	0.221	-	8.4%	0.00072	3.1–13.6	-	-	-	-	-	-
lowly competitive	59	-	209	-	0.220	-	8.2%	0.00143	0.8–15.6	-	-	-	-	-	-
highly competitive	59	-	207	-	0.222	-	8.5%	0.00145	1.0–15.9	-	-	-	-	-	-

Note---*p*_1_ and *p*_2_ denote the probabilities of directing the participant to the sensitive question. The column **a** shows the relative frequency of yes-responses.

For the Wiesbaden subsample surveyed with CDM, the estimates of the prevalence for honest-yes respondents were low for both physical doping (4.2%) and cognitive doping (0.7%) and did not differ from zero. Again, there was no clear sign for a modulation of prevalence rates by competition motivation. As in the Frankfurt sample, the cheater rates were high (physical doping: 54%, cognitive doping: 45%).

For the Wiesbaden subsample surveyed with UQM, the estimate of prevalence *π*_*S*_ for physical doping (3.9%) mimicked the one obtained by CDM. The results of the likelihood-ratio test confirmed this impression, χ^2^(1) = 0.004, *p* = .948. In contrast, the estimated prevalence for cognitive doping (8.4%) was somewhat higher than the one obtained by the CDM. However, the likelihood-ratio test did not provide evidence that the two estimated prevalences significantly differed, χ^2^(1) = 2.690, *p* = .101. Again, the prevalence rates for physical and cognitive doping were virtually identical for lowly and highly competitive participants.

To assess whether prevalences estimated by UQM changed significantly from the survey conducted in 2011, we compared the present estimates in the Wiesbaden UQM subsample with the corresponding estimates obtained in 2011, which were 9.7% for physical doping and 13.2% for cognitive doping [6]. Although the present prevalence rates were numerically smaller than the previous one, likelihood-ratio tests did not indicate a significant change; physical doping: χ^2^(1) = 2.844, *p* = .092; cognitive doping: χ^2^(1) = 1.827, *p* = .177.

## Discussion

The present survey focused on three objectives. The first objective was whether UQM and CDM would provide similar 12-month prevalence estimates of physical and cognitive doping. We did not observe meaningful differences for prevalence rates estimated by CDM and UQM in the Wiesbaden sample. This suggests that both procedures can provide converging results. The second objective was whether the level of competition motivation would modulate the prevalences for physical and cognitive doping. We did not observe any evidence for such an influence. This result strengthens the notion of a previous study [19] that competiveness does not predict doping behavior. Finally, we were interested whether the prevalences of physical and cognitive doping have changed since the last survey conducted in 2011 [6]. Although the prevalences for both physical and cognitive doping were somewhat smaller in the present survey, they did not decrease significantly. To the best of our knowledge, there are no other studies available that assessed the prevalence of cognitive doping in athletes. Compared to studies investigating the use of cognitive-enhancing substance use in students [23], surgeons [24], as well as readers of the journals “Nature” [25] and “Handelsblatt” [26], which all showed prevalences around 20%, the present survey in triathletes indicates somewhat smaller prevalences. However, keeping in mind that the cheater rate in the present survey for the question regarding cognitive doping was higher than 60%, the true rate of cognitive dopers might be underestimated.

Particularly striking are the high estimates of cheaters obtained with CDM compared to a previous study [9]. The present estimates were quite similar for both sensitive questions about physical and cognitive doping and also for the Frankfurt and Wiesbaden samples. This specific result indicates that most of the participants did not provide honest responses and tended to respond with “no”. There are two extreme alternative explanations. First, one could argue that all cheaters are dopers. This would mean that up to 75% of all athletes were dopers in the present survey. Second, one could argue that all dopers answered truthfully and that all cheaters represent non-dopers, who refrain from saying “yes”. In this case, the true prevalence would be equal to the estimated rate of honest yes-respondents (i.e., *π*_*S*_). Certainly, neither of these two extreme views is particularly realistic. Contrary, *π*_*S*_ and *π*_*S*_ + *γ* can be regarded as lower and upper bounds, respectively, of the proportion of doping [13].

It should be kept in mind that UQM is not able to identify cheating. Nevertheless, when a doper has to answer the sensitive question, he or she may still respond with no. In this case, the prevalence rate of UQM would also underestimate the true prevalence. Therefore, a disadvantage of UQM is that it does not provide an upper bound other than 100%. In addition, about 25% of the surveyed athletes did not fully complete the questionnaire. Many of these athletes denied answering at least one of the two sensitive questions. It seems reasonable to assume that the proportion of dopers within this group of athletes is especially high, which would also imply an underestimation of the true prevalence rate in the present study.

In sum, the present survey on recreational triathletes supports the idea that UQM and CDM can provide similar estimates of doping behavior. Nevertheless, the CDM also suggests that these estimates underreport the true doping behavior, that is, the estimates only provide a lower bound. Moreover, the present results strengthen the notion that the level of competiveness does not predict doping behavior. Finally, the present results are consistent with the results reported in the 2011 survey. The prevalence estimates in the present study are numerically but not significantly lower. Clearly, these results indicate that doping behavior is a problem not only in elite athletes but also in recreational sports and that the portion of dopers among recreational athletes is stable at least within a time window of three years. Future research may investigate doping behavior not only in endurance disciplines but also in popular team sports like soccer. Furthermore, follow-up studies are necessary to investigate whether current anti-doping programs such as the World Anti-Doping Agency’s (WADA) initiative of values-based education programs will result in lower doping prevalences not only in elite’s sports but also in recreational sports.

## Supporting Information

S1 File Dataset. (SAV)

S2 File Coding dataset. (PDF)

## References

[1] Simon P, Striegel H, Aust F, Dietz K, Ulrich R. Doping in fitness sports: estimated number of unreported cases and individual probability of doping. Addiction. 2006;101: 1640–1644. 10.1111/j.1360-0443.2006.01568.x 17034444

[2] Dietz P, Ulrich R, Niess A, Best R, Simon P, Striegel H. Prediction profiles for nutritional supplement use among young german elite athletes. Int J Sport Nutr Exerc Metab. 2014;24: 623–631. 10.1123/ijsnem.2014-0009 24901677

[3] Striegel H, Ulrich R, Simon P. Randomized response estimates for doping and illicit drug use in elite athletes. Drug Alcohol Depend. 2010;106: 230–232. 10.1016/j.drugalcdep.2009.07.026 19740612

[4] Hon O de, Kuipers H, Bottenburg M van. Prevalence of Doping Use in Elite Sports: A Review of Numbers and Methods. Sport Med. 2014; 1–13. 10.1007/s40279-014-0247-x 25169441

[5] Striegel H, Simon P, Frisch S, Roecker K, Dietz K, Dickhuth HH, et al Anabolic ergogenic substance users in fitness-sports: A distinct group supported by the health care system. Drug Alcohol Depend. 2006;81: 11–19. 10.1016/j.drugalcdep.2005.05.013 16009506

[6] Dietz P, Ulrich R, Dalaker R, Striegel H, Franke AG, Lieb K, et al Associations between physical and cognitive doping—A cross-sectional study in 2.997 triathletes. PLoS One. 2013;8 10.1371/journal.pone.0078702 PMC3827233 24236038

[7] Franke AG, Bagusat C, Rust S, Engel A, Lieb K. Substances used and prevalence rates of pharmacological cognitive enhancement among healthy subjects. Eur Arch Psychiatry Clin Neurosci. 2014;264: 83–90. 25214391 10.1007/s00406-014-0537-1

[8] Chaudhuri A, Christofides TC. Indirect questioning in sample surveys. Heidelberg: Springer; 2013.

[9] Pitsch W, Emrich E, Klein M. Doping in elite sports in Germany: results of a www survey. Eur J Sport Soc. 2007;4: 89–102.

[10] Lensvelt-Mulders GJLM. Meta-Analysis of Randomized Response Research: Thirty-Five Years of Validation. Sociol Methods Res. 2005;33: 319–348. 10.1177/0049124104268664

[11] Warner SL. Randomized response: a survey technique for eliminating evasive answer bias. J Am Stat Assoc. 1965;60: 63–66. 12261830

[12] Greenberg BG, Abul-Ela A-LA, Simmons WR, G.Horvitz D. The Unrelated Question Randomized Response Model: Theoretical Framework. J Am Stat Assoc. 1969;64: 520–539.

[13] Clark SJ, Desharnais RA. Honest answers to embarrassing questions: Detecting cheating in the randomized response model. Psychol Methods. 1998;3: 160–168.

[14] Pitsch W. Minimizing response bias: an application of the randomized response technique In: Barkoukis V, Lazuras L, Tsorbatzoudis H, editors. The psychology of doping in sport. London: Routledge; 2016 pp. 111–125.

[15] Blair G, Imai K, Zhou Y-Y. Design and Analysis of the Randomized Response Technique. J Am Stat Assoc. 2015;110: 1304–1319. 10.1080/01621459.2015.1050028

[16] Ulrich R, Schröter H, Striegel H, Simon P, Schro H, Schroter H. Asking sensitive questions: a statistical power analysis of randomized response models. Psychol Methods. 2012;17: 623–641. 10.1037/a0029314 22924599

[17] Moran A, Guerin S, Kirby K, MacIntyre T. The development and validation of a doping attitudes and behaviour scale: Report to World Anti-Doping Agency and The Irish Sports Council. 2008.

[18] Waldron JJ, Krane V. Whatever it Takes: Health Compromising Behaviors in Female Athletes. Quest. 2005;57: 315–329. 10.1080/00336297.2005.10491860

[19] Petróczi A. Attitudes and doping: a structural equation analysis of the relationship between athletes’ attitudes, sport orientation and doping behaviour. Subst Abus Treat Prev policy. 2007;2: 35 10.1186/1747-597X-2-34 PMC2217289 17996097

[20] Schuler H, Thornton GCI, Frintrup A, Mueller-Hanson R. Achievement Motivation Inventory (AMI). Göttingen, Bern, New York: Hans Huber; 2002.

[21] Schuler H, Prochaska M. Leistungsmotivationsinventar. Göttingen: Hogrefe; 2001.

[22] Mood AM, Graybill FA, Boes DC. Introduction to the Theory of Statistics. New York: McGraw-Hill; 1974.

[23] Dietz P, Striegel H, Franke AG, Lieb K, Simon P, Ulrich R. Randomized response estimates for the 12-month prevalence of cognitive-enhancing drug use in university students. Pharmacotherapy. 2013;33: 44–50. 10.1002/phar.1166 23307544

[24] Franke AG, Bagusat C, Dietz P, Hoffmann I, Simon P, Ulrich R, et al Use of illicit and prescription drugs for cognitive or mood enhancement among surgeons. Bmc Med. 2013;11: 102 10.1186/1741-7015-11-102 23570256 PMC3635891

[25] Maher B. Poll results: look who’s doping. Nature. 2008;452: 674–675. 10.1038/452674a 18401370

[26] Dietz P, Soyka M, Franke AG. Pharmacological Neuroenhancement in the Field of Economics—Poll Results from an Online Survey. Front Psychol. 2016;7: 520 10.3389/fpsyg.2016.00520 27148128 PMC4835716

