# Supplementary material for: A Comparison of the Cheater Detection and the Unrelated Question Models: A Randomized Response Survey on Physical and Cognitive Doping in Recreational Triathletes
Source: PLoS One. 2016 May 24;11(5):e0155765. doi: 10.1371/journal.pone.0155765 (PMC4878800; doi:10.1371/journal.pone.0155765)
Supplement: S2 File — (PDF) [file pone.0155765.s002.pdf]

Number	Variable	Definition, Coding
01	LikertSumCM	Competition motivation score<br><br>Possible range: 10-70 (Likert sum)
02	AnswerRRTCognitive	Answer to RRT procedure concerning cognitive doping<br><br>0: no<br>1: yes
03	AnswerRRTPhysical	Answer to RRT procedure concerning physical doping<br><br>0: no<br>1: yes
04	Gender	Gender<br><br>0: male<br>1: female
05	Age	Age (years)
06	BodyHeight	Body height (cm)
07	BodyWeight	Body weight (cm)
08	YearsTraining	Years of training experience (years)
09	HoursTrainingWeek	Hours of training/week (hours)
10	ALevels	A-levels<br><br>0: no<br>1: yes
11	KmTrainingSwimming	Training kilometers/week (km), swimming
12	KmTrainingBiking	Training kilometers/week (km), biking
13	KmTrainingRunning	Training kilometers/week (km), running
14	GroupTraining	Athlete mainly trains in training group<br><br>0: no<br>1: yes

Number	Variable	Definition, Coding
15	SoloAthlete	Athlete participates in the triathlon event as a solo athlete<br><br>0: no (athlete is a member of a relay team)<br>1: yes
16	Location	Location of triathlon event<br><br>0: Frankfurt<br>1: Wiesbaden
17	LanguageQuestionnaire	Language of questionnaire (selected by athlete)<br><br>0: German<br>1: Englisch
18	RRTModel	RRT model<br><br>0: Cheater Detection Model (CDM)<br>1: Unrelated Question Model (UQM)
19	ProbSensQuestCog	Probability to be directed to the sensitive question about cognitive doping<br><br>0: 0.25 (only CDM)<br>1: 0.75 (only CDM)<br>3: 0.67 (only UQM)
20	ProbSensQuestPhys	Probability to be directed to the sensitive question about physical doping<br><br>0: 0.25 (only CDM)<br>1: 0.75 (only CDM)<br>3: 0.67 (only UQM)
21	RRTOrder	Order of RRT procedures<br><br>0: cognitive doping first, physical doping second<br>1: physical doping first, cognitive doping second
22	CaseExcluded	Case excluded from final data analysis<br><br>0: no (case included in final data analysis)<br>1: yes, because of missing data<br>2: yes, because of outlier rejection<br>3: yes, because of visual inspection
23	CompMotiv	Competition motivation score above the median (separately for Frankfurt-CDM, Wiesbaden-CDM, and Wiesbaden-UQM; only included cases)<br><br>0: no (lowly competitive)<br>1: yes (highly competitive)
24	BodyMassIndex	Body mass index (kg/m^2; only included cases)
